# Supplementary material for: An open-source framework for large-scale, flexible evaluation of biomedical text mining systems
Source: J Biomed Discov Collab. 2008 Jan 29;3:1. doi: 10.1186/1747-5333-3-1 (PMC2276192; doi:10.1186/1747-5333-3-1)
Supplement: Additional file 1 — Evaluation platform source code. The additional file contains the software evaluation framework discussed in this paper. The most current release can be downloaded from SourceForge.net [1]. See the accompanying README file for instructions on installation and use. Also included in the distribution is a collection of UIMA wrappers for some commonly used BioNLP tools and annotated corpora. [file 1747-5333-3-1-S1.zip › bionlp_uima_repository-1.0.1/docs/api/overview-summary.html]

Overview (Center for Computational Pharmacology Util/UIMA API)


|  |  |  |
| --- | --- | --- |
| **Overview** \| Package \| Class \| **Tree** \| **Deprecated** \| **Index** \| **Help** | |  |
| PREV   NEXT | **FRAMES**    **NO FRAMES**     **All Classes** |  |


---


# Center for Computational Pharmacology Util/UIMA API

| **Packages** | |
| --- | --- |
| **edu.uchsc.ccp.uima.ae.nlp.anaphora** |  |
| **edu.uchsc.ccp.uima.ae.nlp.ei.gene** |  |
| **edu.uchsc.ccp.uima.ae.nlp.ei.mutation** |  |
| **edu.uchsc.ccp.uima.ae.nlp.opendmap** |  |
| **edu.uchsc.ccp.uima.ae.nlp.parser** |  |
| **edu.uchsc.ccp.uima.ae.nlp.sentenceDetector** |  |
| **edu.uchsc.ccp.uima.ae.nlp.stemmer** |  |
| **edu.uchsc.ccp.uima.ae.nlp.tokenizer** |  |
| **edu.uchsc.ccp.uima.ae.util** |  |
| **edu.uchsc.ccp.uima.ae.util.converter** |  |
| **edu.uchsc.ccp.uima.ae.util.filter** |  |
| **edu.uchsc.ccp.uima.annotation** |  |
| **edu.uchsc.ccp.uima.annotation.syntactic** |  |
| **edu.uchsc.ccp.uima.cc.summary** |  |
| **edu.uchsc.ccp.uima.cc.summary.comparison** |  |
| **edu.uchsc.ccp.uima.cc.summary.opendmap** |  |
| **edu.uchsc.ccp.uima.mention** |  |
| **edu.uchsc.ccp.uima.util** |  |
| **edu.uchsc.ccp.uima.util.opendmap** |  |
| **edu.uchsc.ccp.util** |  |
| **edu.uchsc.ccp.util.external.abgene** |  |
| **edu.uchsc.ccp.util.external.abner** |  |
| **edu.uchsc.ccp.util.external.geniatagger** |  |
| **edu.uchsc.ccp.util.external.kex** |  |
| **edu.uchsc.ccp.util.external.lingpipe** |  |
| **edu.uchsc.ccp.util.external.opennlp** |  |
| **edu.uchsc.ccp.util.external.penn** |  |
| **edu.uchsc.ccp.util.external.stanfordparser** |  |
| **edu.uchsc.ccp.util.nlp** |  |
| **edu.uchsc.ccp.util.nlp.annotation** |  |
| **edu.uchsc.ccp.util.nlp.annotation.comparison** |  |
| **edu.uchsc.ccp.util.nlp.annotation.filter** |  |
| **edu.uchsc.ccp.util.nlp.annotation.printer** |  |
| **edu.uchsc.ccp.util.nlp.document** |  |
| **edu.uchsc.ccp.util.nlp.ei** |  |
| **edu.uchsc.ccp.util.nlp.ei.xml** |  |
| **edu.uchsc.ccp.util.nlp.mention** |  |
| **edu.uchsc.ccp.util.nlp.mention.comparison** |  |
| **edu.uchsc.ccp.util.nlp.parser.corpus** |  |
| **edu.uchsc.ccp.util.nlp.stats** |  |
| **edu.uchsc.ccp.util.nlp.stemmer** |  |
| **edu.uchsc.ccp.util.nlp.tokenizer** |  |
| **edu.uchsc.ccp.util.xml** |  |

---


|  |  |  |
| --- | --- | --- |
| **Overview** \| Package \| Class \| **Tree** \| **Deprecated** \| **Index** \| **Help** | |  |
| PREV   NEXT | **FRAMES**    **NO FRAMES**     **All Classes** |  |


---
